# Participant diversity is necessary to advance brain aging research

**DOI:** 10.1016/j.tics.2023.12.004

**Published:** 2024-01-27

**Authors:** Gagan S. Wig, Sarah Klausner, Micaela Y. Chan, Cameron Sullins, Anirudh Rayanki, Maya Seale

**Affiliations:** 1 Center for Vital Longevity, The University of Texas at Dallas, Dallas, TX 75235, USA; 2 Department of Psychology, School of Behavioral and Brain Sciences, The University of Texas at Dallas, Dallas, TX 75080, USA; 3 Department of Psychiatry, The University of Texas Southwestern Medical Center, Dallas, TX 75390, USA; 4 https://www.wigneurolab.org

## Abstract

An absence of population-representative participant samples has limited research in healthy brain aging. We highlight examples of what can be gained by enrolling more diverse participant cohorts, and propose recommendations for specific reforms, both in terms of how researchers accomplish this goal and how institutions support and benchmark these efforts.

More individuals are reaching older age adulthood than ever before. The average life expectancy of a child born today is close to 80 years; by 2050, the global population of individuals over age 60 years will nearly double from 12% to 22% [1]. However, living longer means we are more prone to the wear, tear, and diseases of advanced age. Age is the biggest risk factor for dementia; after age 65 years, an individual’s risk of Alzheimer’s disease (AD) doubles every 5 years [2]. Even in the absence of disease, cognitive abilities decline during adulthood and this decline accelerates in later decades [3]. However, not everyone ages the same: some individuals remain cognitively healthy into their 90s, while others begin to experience cognitive decline in middle age. Given the immense variability in how our cognitive abilities change with age, how can we best uncover the mysteries of brain aging? Understanding the causes and consequences of age-related brain changes necessitates studying individuals who vary in their aging trajectories. However, current practices in brain aging research have inadvertently imposed barriers to participation, resulting in research cohorts comprised of participants who are racially homogeneous (primarily White) and more educated, affluent, and healthy than the general population. The relative absence of participant samples that are more representative of the population has undermined scientific progress, not only by limiting the generalizability of findings but by confining descriptions of brain aging to those provided by a narrow subset of the population who are less likely to experience the typical burdens of advancing age.

## Not just more people, but more types of people

There is well-placed attention towards increasing participant sample sizes to improve measurement reliability in human neuroscience research [4]. While this emphasis is important, focusing scientific efforts on maximizing sample size without ensuring population representative participant composition can restrict our understanding of the mechanisms of cognitive processing and the sources of phenotypic variation across individuals [5].

In AD research, inclusion of more representative samples have provided evidence for differences in the health and lifestyle risk factors [6] and etiology and pathogenesis [7] of dementia among racial and ethnic minorities compared to non-Hispanic White participants, challenging the generality of several prevailing assumptions. Meanwhile, an absence of population representative patient cohorts in clinical trials has hampered evaluation of the safety and generalizability of AD drug treatments [8]. These observations should serve as a lesson for brain aging research more broadly.

If the goal is to understand how and why the brain ages over time, we must study individuals from a wider range of the health continuum of aging. Homogeneous samples of healthy and privileged aging cohorts can inform our understanding of what the aging brain can look like in ideal circumstances, but this approach severely constrains our understanding of the range of aging-related brain changes, and it does not provide an accurate description of the reality experienced by most individuals.

Critically, the challenges that limit participation in brain aging research are not experienced uniformly across the population. Rather, they disproportionately affect individuals of lower socioeconomic status and ethnic and racial minorities. The burdens of accelerated and pathological brain aging are also more prevalent in these same cohorts of individuals [9]. There is under-representation of large segments of the population that are at highest risk for aging-related cognitive decline and dysfunction, and who occupy an important and informative segment of the distribution of brain aging variation.

**Figure 1.**
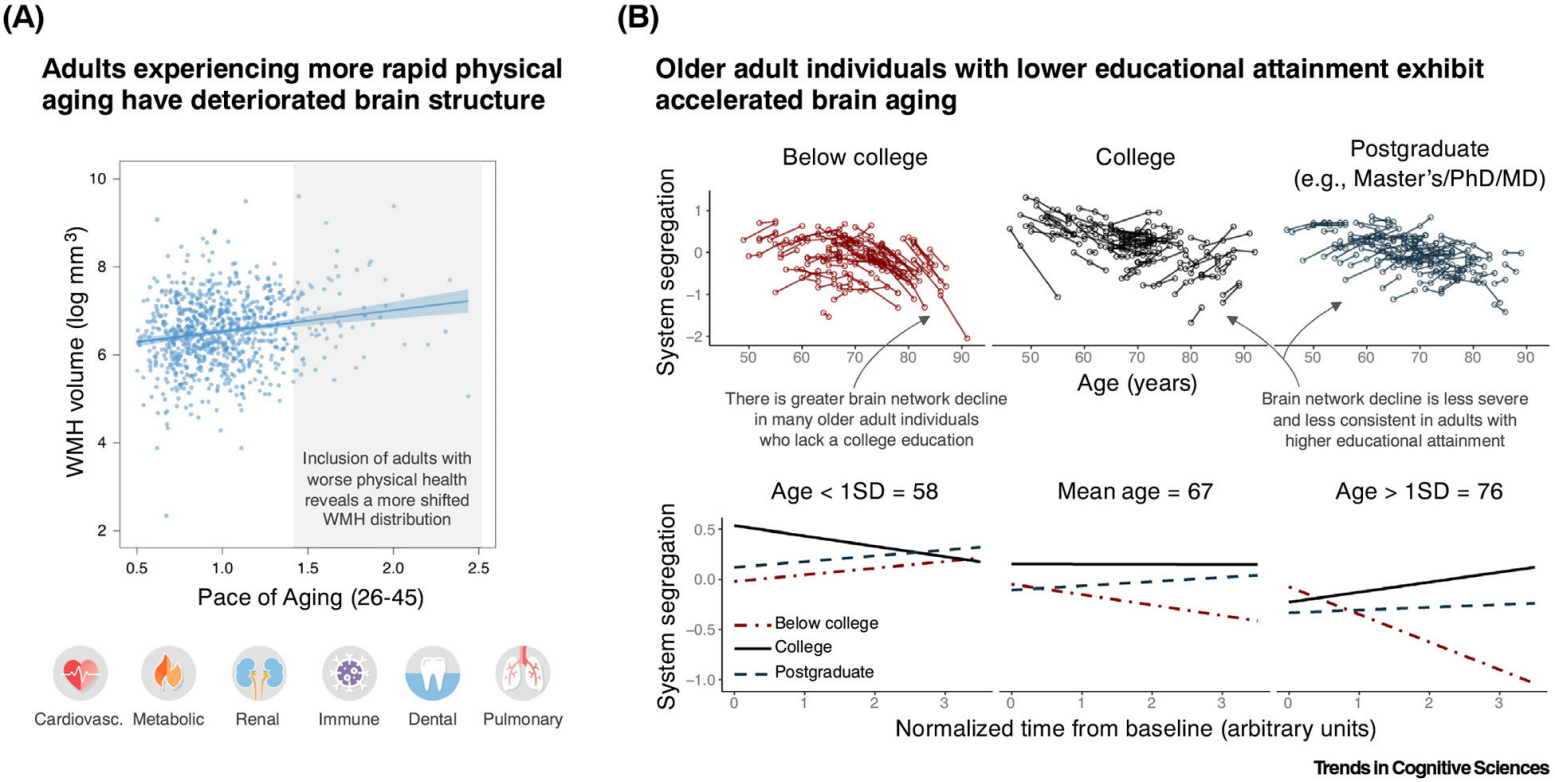
Including more population representative cohorts of individuals leads to novel insights regarding how the brain ages. (A) Individuals’ volume of white matter hyperintensities in the brain (WMH; measured at age 45 years) plotted in relation to a measure of their multiorgan physical aging (pace of aging, measured over multiple occasions from 26 to 45 years; *n* = 851). Individuals that exhibit more accelerated rates of physical aging exhibit worse white matter health at age 45 years (both indicated by higher values). The study cohort represents a wide range of socioeconomic status and key health indicators for individuals of the same age. Each dot corresponds to one participant. Pace of aging was derived from health indicators related to the functioning of multiple organ systems (lower images). Adapted from [10]. (B) Top: longitudinal changes in resting-state functional brain network organization (system segregation) are plotted as a function of educational attainment among adult individuals (*n* = 265, baseline age 45–86 years, 154 females). Each line corresponds to an individual’s changes in brain system segregation, estimated longitudinally (two to five occasions and ranging over 1–9 years, depending on the individual). Bottom: simple slope plots of average system segregation change scores for three representative ages. The figure panels demonstrate that there is variability in the magnitude and timing of brain network changes across adulthood. However, a significant portion of this variability can be explained by the absence of a college education, particularly among older adult individuals (~65+ years), whereby individuals lacking a college education exhibit accelerated brain network decline. College education was related to other individual- and community-level social determinants of health (not depicted). System segregation is a measure of the functional specialization of the brain network. Lower brain system segregation is linked with worse cognitive ability and alterations in brain activity, and longitudinal declines in system segregation are prognostic of dementia. Adapted from [11]. The inclusion of participants with wider-ranging levels of physical health trajectories and varying life course exposures (the latter indexed by educational attainment) leads to a more accurate estimation of the distribution of brain aging across the population, and reveals distinct patterns and trajectories of brain aging across individuals. Identifying individuals who age in different and tractable ways is an important step towards identifying the causes and consequences of age-related brain decline.

Including participants who vary in racial and ethnic backgrounds, socioeconomic status, physical health, and life course exposures provides a more nuanced and accurate perspective of the brain aging continuum. These sources of participant variability can also be directly interrogated as variables of interest to better chart normative trajectories of brain aging and isolate the determinants of their differences (Figure 1).

## Recognizing the sources of the problem

Biases in participant inclusion are not unique to studies of brain aging, and several important articles have highlighted the need for population representation in human neuroscience research (see Supplemental information online for an extended reading list, emphasizing issues related to older adult cohorts). However, research on healthy adult brain aging poses unique challenges.

First, consider the typical circumstances under which aging adult participants are enrolled in neuroimaging studies. There are physical constraints that prohibit participation in studies that implement certain neuroimaging technologies (e.g., MRI-incompatible implants, claustrophobia, or mobility or size impediments). Many of these impediments are more prevalent in middle-aged and older adults and are particularly characteristic of aging individuals with reduced and declining health.

Second, brain aging studies typically adopt a broad set of participant exclusion criteria that are motivated by the research questions. Commonly, in studies characterizing healthy adult participants (as a control group or as a focus of a study), individuals’ psychiatric, neurological, and physical health history are surveyed before enrollment to evaluate study eligibility. The exclusion criteria are meant to minimize sources of variability that may confound results. While this approach is often reasonably justified, the exclusion criteria can be unnecessarily restrictive, resulting in missed opportunities to improve study validity without compromising study goals. Furthermore, as the likelihood and diagnosis of psychiatric and neurological disorders and chronic physical health conditions increases with age [12,13], the pool of healthy older adults, as defined by highly restrictive criteria, is diminishingly small relative to the population. Consequently, most healthy aging adults enrolled in neuroimaging experiments are unrepresentative of the general population, who have not had the privilege of experiencing flawless health throughout their lifetime.

Third, in studies of healthy aging, cognitive screening tools are often incorporated to minimize the likelihood of enrolling individuals with dementia or severe cognitive impairment. However, these screeners can disproportionately disqualify lower educated individuals, racial minorities, and those whose primary spoken language differs from the testing material [14,15], even when these individuals are cognitively unimpaired.

Critically, the absence of population representative aging adult participant samples is not entirely due to these exclusion criteria. Convenience sampling is a common sampling approach that involves recruiting participants from an easily accessible and engageable pool of the population. This process typically results in nonrepresentative participant cohorts, given that the factors that lead to access and engagement in research are often selective to specific population segments. Brain aging research is particularly prone to this source of biased recruitment given the amplification of the factors which restrict awareness and involvement in research among aging communities. Defaulting to convenience sampling is avoidable but requires setting a clear goal for the intended study cohort (e.g., recruiting a participant sample that matches the census-based demographic, socioeconomic, and health makeup of the study population) and incorporating procedures that increase enrollment of these individuals.

Collectively then, many common research recruitment and testing practices in brain aging research result in pervasive and often unrecognized inclusion barriers (Table 1).

## How to increase population representativeness in brain aging research

Increasing population representativeness first necessitates identifying sources of bias stemming from an established research culture, and then engaging in practices to overcome these barriers. As can be appreciated from Table 1, these reforms require time, effort, and resources. Several larger (and often multisite) research initiatives have successfully incorporated some of the listed practices in their projects. It is often assumed that these are exceptional cases, where the large number of enrolled participants allows for diversity and can buffer unanticipated variability. But more conventional-scale scientific efforts can also achieve participant cohorts that are representative of the local population demographics, without compromising their scientific questions.

Importantly, the responsibility for reform is not limited to researchers. Institutional stakeholders must facilitate efforts towards increasing participant diversity. Universities and hospitals play major roles in the communities they serve and have extensive reach to broad segments of the community (via education, healthcare, sports, and arts). This reach should be leveraged to encourage research engagement. Public and private funding institutions have begun to commit financial resources to support broader recruitment efforts, but they should also take steps to remove constraints that disincentivize efforts to increase population representativeness in participant cohorts. Researchers are often unable to devote the time needed to recruit diverse participant samples as they are bound by project timelines that make this goal unfeasible. Consequently, convenience-based recruitment becomes necessary to accomplish study aims within allotted time windows, at the cost of enrolling cohorts that are representative of the community’s racial, ethnic, economic, and health demographics. If the latter is valued, institutional provision and tolerance are necessary.

## Concluding remarks

Research on brain aging has been hindered by a lack of population representative participant cohorts. This limitation is being confronted in patient-based research, but also extends to research across a broader continuum of healthy brain aging. An absence of efforts to minimize barriers to participation results in a science biased towards characterizing brain aging in extremely healthy and privileged individuals. This bias restricts efforts to isolate the mechanisms of brain aging, impedes understanding of the factors that confer vulnerability and resilience to age-accompanied decline, and marginalizes individuals who are at the greatest risk of age-related brain disease from being included in research efforts. Investment of resources and adoption of research reforms at the laboratory and institutional level can overcome these limitations and will accelerate the science of the aging brain.

## Supplementary Material

supplementary reading list

## Figures and Tables

**Table 1.** How to overcome barriers to achieving population representation in brain aging research

Participant and study challenges	Solutions and accommodations
Time constraints due to work obligations or limited social/financial support (e.g., childcare assistance)	• Offer research testing appointments during evenings or weekends. • Divide lengthy appointments into multiple shorter sessions, distributed across days if possible. • Provide childcare options at or near the research facility.
Physical distance from research center/lack of transportation	• Provide transportation reimbursement (e.g., mileage allowance) or offer transportation using ride-hailing services in areas where public transportation is limited. • Use satellite research campuses. • Offer testing virtually, via web or smartphones, or in-person at participant’s home (e.g., mobile testing services). The incorporation of alternate testing mediums requires researchers to ensure that the tests are valid and comparable to other forms of testing, to avoid introduction of confounding factors.
Lack of awareness of opportunities to participate	• Advertise study in diverse geographical locations to recruit individuals from a broader range of communities (e.g., spanning income levels, rural and urban neighborhoods, etc.). • Use a variety of advertising mediums to increase reach of recruitment [e.g., social media, radio, print (physical and digital), signage in public locations]. • Form relationships with community leaders and organizations who can help raise awareness about research opportunities. • Continuously monitor recruitment progress to gain insights on what methods are working best and adapt recruitment methods accordingly.
Lack of reliable communication channels to schedule, confirm appointment reminders, etc.	• Obtain and utilize multiple communication mediums (e.g., phone, email, text, messaging); expand beyond phone or email by including social-media-based calling and messaging. • Request participant to provide alternate points of contact (e.g., co-workers, friends, family members).
Mistrust in research and/or medical and scientific organizations	• Validate, address, and empathize with the participant’s feelings of mistrust/concerns throughout both the recruitment process but also during study procedures. Minimize use of jargon throughout recruitment and study procedures. • Employ and train research staff and scientists who are socioeconomically, racially, ethnically, and culturally representative of the participant population. • Employ bilingual research assistants to help communicate study goals, procedures, and methods to non-English speaking participants. • Minimize power dynamics (e.g., remind participants that research testing is optional, and that their comfort and safety takes precedence over research testing requirements). • Express cultural sensitivity. • Provide opportunities for community members to learn more about the research they are contributing to (e.g., public lectures at community events). • Disseminate study updates and findings in a platform accessible and comprehensible to the general public and community organizations. • Align community interests in research by collaborating with community members in the development of study methods and goals.
Physical and health-related impediments that result in direct or indirect participant exclusion	• Conduct comprehensive health screening to assess device compatibility (e.g., BMI, MRI compatibility, claustrophobia) before scheduling participants so that physical barriers to participating can be anticipated and potentially accommodated in advance of testing session. • Relax exclusion criteria during health screening to allow individuals with poorer health status/history to participate. • Adopt procedures or use devices (e.g., mock-MRI scanners, larger bore MRI scanners) that acclimate participants to study environment and/or accommodate physical limitations. • Communicate with participant’s primary care physician to assess and confirm suitability (e.g., to evaluate whether implanted devices are MRI compatible). If a participant does not have a primary care physician, take time to carefully review the participant’s medical history with a doctor or MRI technicians on your team to evaluate eligibility. Alternately or in addition, establish a network of healthcare resources for participants by forming collaborations with local clinics and community health centers that could provide participants with access to medical consultations.
Prone to being unfairly disadvantaged on cognitive screening tests	• Adopt cognitive screeners that are not biased towards higher educational attainment or require specific language fluency. • Adjust cognitive screening criteria to account for racial/ethnic minority group differences or educational differences.
Minimizing data loss and impacts of last-minute cancellations	• Adapt procedures to accommodate phenotypic differences that might impede or affect data collection (e.g., using electroencephalogram electrodes designed for coarse hair, training researchers to work with and measure a range of hair types, accounting for the effect of melanin when using functional near-IR spectroscopy). • Utilize specialized hardware and software to minimize data loss that may be higher in certain cohorts due to greater physical discomfort during data collection (e.g., real-time motion monitoring, customized head molds, real-time prospective motion correction). • Allow for more data collection time during testing sessions. • Send frequent appointment reminders using multiple communication channels. • Identify backup participants in case of last-minute cancellations.
Lower likelihood of retention in interventional and longitudinal studies (nonrandom attrition)	• Obtain and utilize multiple communication channels and obtain alternate points of contact (see above), to increase likelihood of successful follow-up communication. • Allocate greater funds to transportation costs for cases where participant relocates to distant locations outside of original recruitment region. • Relax exclusion criteria, to the extent that is possible and safe, for follow-up testing sessions to allow for study completion. • Consider protocol modifications that are more flexible, and allow returning participants to engage in specific study segments based on changes in their health and cognitive status. • Establish feedback sessions aimed at assessing participants’ experiences during testing, addressing any concerns they may have, and providing a process for gaining insights that can improve study procedures during follow-up visits. • Share personalized data summaries, progress charts, or visual representations of the data collected during interim periods of the study.
