# Supplementary material for: Participant diversity is necessary to advance brain aging research
Source: Trends Cogn Sci. Author manuscript; Available in PMC 2024 Mar 8. (PMC10922549; doi:10.1016/j.tics.2023.12.004)
Supplement: supplementary reading list [file NIHMS1953970-supplement-supplementary_reading_list.pdf]

Participant diversity is necessary to advance brain aging research

Supplemental reading list

Gagan S. Wig[1,2,3], Sarah Klausner[1], Micaela Y. Chan[1], Cameron Sullins[1], Anirudh Rayanki[2], Maya Seale[2]

[1]Center for Vital Longevity, The University of Texas at Dallas, Dallas, TX, 75235, USA
[2]Department of Psychology, School of Behavioral and Brain Sciences, The University of Texas at Dallas, Dallas, TX, 75080, USA
[3]Department of Psychiatry, The University of Texas Southwestern Medical Center, Dallas, TX, 75390, USA

General commentaries

Adkins-Jackson, P. B., Kraal, A. Z., Hill-Jarrett, T. G., George, K. M., Deters, K. D., Besser, L. M., Avila-Rieger, J. F., Turney, I., & Manly, J. J. (2023). Riding the merry-go-round of racial disparities in ADRD research. *Alzheimer's & Dementia*, 19(10), 4735–4742. doi: 10.1002/alz.13359

Brewster, P., Barnes, L., Haan, M., Johnson, J. K., Manly, J. J., Nápoles, A. M., Whitmer, R. A., Carvajal-Carmona, L., Early, D., Farias, S., Mayeda, E. R., Melrose, R., Meyer, O. L., Zeki Al Hazzouri, A., Hinton, L., & Mungas, D. (2019). Progress and future challenges in aging and diversity research in the United States. *Alzheimer's & Dementia*, 15(7), 995–1003. doi: 10.1016/j.jalz.2018.07.221

Dotson, V. M., & Duarte, A. (2020). The importance of diversity in cognitive neuroscience. *Annals of the New York Academy of Sciences*, 1464(1), 181-191. doi: 10.1111/nyas.14268

Ghai, S. (2021). It's time to reimagine sample diversity and retire the WEIRD dichotomy. *Nature Human Behavior*, 5, 971–972. doi: 10.1038/s41562-021-01175-9

Henrich, J., Heine S. J., & Norenzayan, A. (2010). The weirdest people in the world? *Behavioral and Brain Sciences*, 33(2-3), 61-83. doi: 10.1017/S0140525X0999152X

Ricard, J. A. *et al.* (2023). Confronting racially exclusionary practices in the acquisition and analyses of neuroimaging data. *Nature Neuroscience,* 26, 4–11. doi: 10.1038/s41593-023-01516-z

Towfighi, A., Berger, R. P., Corley, A. M., Glymour, M. M., Manly, J. J., & Skolarus, L. E. (2023). Recommendations on social determinants of health in neurologic disease. *Neurology*, 101(7 Supplement 1), S17-S26. doi: 10.1212/WNL.0000000000207562

Practical guides and recommendations

Esiaka, D., Yarborough, C. C., Fausto, B. A., & Gluck, M. A. (2022). A mini-review of strategies for recruiting older African Americans to Alzheimer's disease research. *Community Health Equity Research & Policy*. doi: 10.1177/0272684X221118493

Ejiogu N, Norbeck JH, Mason MA, Cromwell BC, Zonderman AB, Evans MK. (2011). Recruitment and retention strategies for minority or poor clinical research participants: lessons from the Healthy Aging in Neighborhoods of Diversity across the Life Span study. *Gerontologist*, 51:33-45. doi: 10.1093/geront/gnr027.

Eliacin, J., Hathaway, E., Wang, S., O'Connor, C., Saykin, A. J., & Cameron, K. A. (2022). Factors influencing the participation of Black and White Americans in Alzheimer's disease biomarker research. *Alzheimer's & Dementia: Diagnosis, Assessment & Disease Monitoring*, 14(1), e12384. doi: 10.1002/dad2.12384

González, H. M., Tarraf, W., Fornage, M., González, K. A., Chai, A., Youngblood, M., ... & Schneiderman, N. (2019). A research framework for cognitive aging and Alzheimer's disease among diverse US Latinos: Design and implementation of the Hispanic Community Health Study/Study of Latinos—Investigation of Neurocognitive Aging (SOL-INCA). *Alzheimer's & Dementia*, 15(12), 1624-1632. doi: 10.1016/j.jalz.2019.08.192

Hughes, T. B., Varma, V. R., Pettigrew, C., & Albert, M. S. (2017). African Americans and clinical research: evidence concerning barriers and facilitators to participation and recruitment recommendations. *The Gerontologist*, 57(2), 348-358.

Kwasa, J., Peterson, H. M., Karrobi, K., Jones, L., Parker, T., Nickerson, N., & Wood, S. (2023) Demographic reporting and phenotypic exclusion in fNIRS. *Frontiers in Neuroscience*, 17, 1086208. doi: 10.3389/fnins.2023.108620

Loussouarn, G., Garcel, A. L., Lozano, I., Collaudin, C., Porter, C., Panhard, S., Saint-Léger, D., & de La Mettrie, R. (2007). Worldwide diversity of hair curliness: a new method of assessment. *International Journal of Dermatology*, 46, 2–6. doi: 10.1111/j.1365-4632.2007.03453.

Milani S. A., Marsiske M., Cottler L. B., Chen X., & Striley C. W. (2018). Optimal cutoffs for the Montreal Cognitive Assessment vary by race and ethnicity. *Alzheimer's & Dementia: Diagnosis, Assessment & Disease Monitoring*, 10(1), 76-86. doi: 10.1016/j.dadm.2018.09.003

Tzuang, M., Owusu, J. T., Spira, A. P., Albert, M. S., & Rebok, G. W. (2018). Cognitive Training for Ethnic Minority Older Adults in the United States: A Review. *The Gerontologist*, 58(5), e311–e324. doi: 10.1093/geront/gnw260

Weiner, M. W., Veitch, D. P., Miller, M. J., Aisen, P. S., Albala, B., Beckett, L. A., Green, R. C., Harvey, D., Jack, C. R., Jr, Jagust, W., Landau, S. M., Morris, J. C., Nosheny, R., Okonkwo, O. C., Perrin, R. J., Petersen, R. C., Rivera-Mindt, M., Saykin, A. J., Shaw, L. M., Toga, A. W., Tosun, D., Trojanowski, J. Q., & Alzheimer's Disease Neuroimaging Initiative. (2023). Increasing participant diversity in AD research: Plans for digital screening, blood testing, and a community-engaged approach in the Alzheimer's Disease Neuroimaging Initiative 4. *Alzheimer's & Dementia*, 19(1), 307–317. doi: 10.1002/alz.12797
